# Neuronal Plasticity in the Amygdala Following Predator Stress Exposure

**DOI:** 10.3389/fnbeh.2019.00025

**Published:** 2019-02-20

**Authors:** Rupshi Mitra

**Affiliations:** School of Biological Sciences, Nanyang Technological University, Singapore, Singapore

**Keywords:** amygdala, fear, stress, glucocorticoids, predator-prey

## Abstract

Predation causes robust long-term stress-related effects on prey individuals even if they do not get consumed by the predator. Here I review the role of basolateral amygdala (BLA) neurons in the mediation of non-consumptive effects of predation. This brain region is critical for the generation and maintenance of fear response across many phylogenetic groups. The exposure to cues of predator presence activates neurons within the BLA. Hormones secreted during stressful episodes cause long-lasting structural changes in BLA neurons, causing facilitation of endocrine response during subsequent exposure to stressful episodes like later predator exposure. Some studies also suggest that BLA is involved in creating anticipatory defensive behavior in response to the expectation of change in the environment.

## Predator Cues Cause Environment-Dependent Flexibility in Prey Response

Predation has been considered by both ecologists and neurobiologists as a fundamental biological process (Clinchy et al., 2013). The approaches taken by these disciplines somewhat differ. For an ecologist, predation is a consumptive and acute process. A predator stalks a prey. The encounter evokes an immediate fight-or-flight response. A chase ensues. Prey is either eaten up, contributing to mortality rates; or the victim escapes to partake in other processes of life like reproduction and foraging. Mortality rates can then be modeled to predict population dynamics for predator-prey dyads, and so on. The crucial thing here is the assumption that the predation does not lead to an effect on prey beyond the encounter itself. Effects of predation can be summed up by consumptive rates of capture. Can the elk population in Yellowstone be modeled once wolves are introduced? Can we predict cyclicity of wolf population as a function of prey density? These sorts of questions dominate the to-do list of an ecologist. For a neurobiologist, however, predation is very much a non-consumptive process. Prey animals in this narrative develop a fear of cues of predator presence, like urine or body odors. These signals initiate an innate aversion that reduces the chance of a predator encounter. The fear can be generalized by the process of association whereby previously emotion-neutral environmental stimuli gain emotional valence (“Avoid that waterhole because last time I smelled a predator there”). Fear causes chronic effects like lower investment in reproductive hormones, greater activation of the stress axis and lower growth rates. Thus, the impact of predation includes chronic effects of fear itself beyond the direct mortality of the prey. Which brain regions should be lesioned to observe less fear of predators? Which molecules should be over-expressed to increase fear? These sort of questions are a mainstay for a neurobiologist of fear. So, in short, analysis of predation takes the form of acute consumption of one organism by another on the one hand; and chronic non-consumptive proximate changes on the other. This division reflects the difference in scholarly tradition. This division does not reflect the divergence of the underlying biological process itself. Thus, multiple research teams have successfully integrated acute and chronic effects of the predations (Clinchy et al., 2011; Buck and Ripple, 2017; Hermann and Landis, 2017).

There are two inter-related processes that mediate non-consumptive predation effects on the prey. First, coping with predators evoke a fight-or-flight response which requires the initiation of stress endocrine axis (Harris and Carr, 2016). Repeated or prolonged activation of stress endocrine response causes allostatic load on the neuroendocrine mechanism (McEwen, 1998). For example, excessive stress hormones over long-time cause atrophy of hippocampal neurons that provides negative feedback on the release of adrenal hormones, causing an increase in stress hormones and further exacerbating hippocampal atrophy (Magariños and McEwen, 1995). Similarly, excessive adrenal glucocorticoids suppress the immune system which is adaptive during acute phases of stress but become maladaptive during chronic episodes. Non-consumptive effects in this framework are then the unavoidable cost of mounting defense to the predators. A second framework can also be proposed based on predictive regulation of behavior and physiology by the brain. The brain can detect environmental cues of predator presence and then change the physiological landscape in anticipation of need for the higher defense. Thus, environmental adversity can percolate from mother to child through changes in maternal care and enhances stress responsivity (Liu et al., 1997). Alternatively, stress hormones *in utero* can change the growth trajectory of offspring (Dantzer et al., 2013). Non-consumptive effects in this framework reflect environment-dependent plasticity in the prey behavior. Both allostatic load and environment-dependent plasticity converge on the same proximate mechanisms and are thus inter-related.

## The Contribution of Basolateral Amygdala in Mediation of Defensive Behaviors

Amygdala is a collection of structurally heterogeneous brain regions in the medial temporal lobe (Sah et al., 2003). These brain regions are divided into three broad groups that include basolateral, cortical and centromedial amygdala. Basolateral complex amongst these group has been widely studied in the context of fear or conditioning of fearful stimuli to innocuous environmental cues (LeDoux, 1998). Molecular evidence suggests that broad features of amygdala were present in concestor basal to the vertebrates (Martínez-García et al., 2002; Medina et al., 2011). Homologs of basolateral amygdala (BLA) have been found in amphibians and birds, supporting an ancestral origin of this brain structure and its associated functions (Cheng et al., 1999; Moreno and González, 2007).

Exposure to predator cues activate neurons within BLA (Table 1). This has been studied using Fos, an immediate early gene product that is often used as a proxy for recent neuronal activity. Exposing rats to cat odors causes Fos expression in the BLA (Dielenberg et al., 2001). Similarly, exposure to ferret odor leads to recruitment of CaMKII positive neurons suggesting an influx of Ca^2+^ during the odor exposure (Butler et al., 2011). Electrophysiological studies also reveal neurons within BLA selectively fires to exposure of anesthetized rats to cat urine (Karst et al., 2002). All these chemosensory cues cause defensive behavior in rats. Similarly, infection with *Toxoplasma gondii* reduces fear to cat urine in rats and in parallel causes dendritic retraction within BLA neurons (Mitra et al., 2013). This suggests that inflammation associated with infections can influence processing of the fear within BLA. On the other hand, this observation can also be interpreted in view of direct effect of parasite presence within the BLA.

**Table 1:** Role of basolateral amygdala (BLA) in defensive behaviors.

Animal model; and treatments	Observation(s)	References
Wistar rats; Exposure to worn cat collar	Expression of immediate early gene (c-Fos) in BLA; in addition to regions of medial hypothalamic zone	Dielenberg et al. (2001)
Long-Evans rats; Exposure to ferret-scented towel	Increase in BLA neurons colabeled with CaMKII and c-Fos	Butler et al. (2011)
Wistar rats; Acute brain slices washed with glucocorticoid receptor agonist	Increase in voltage-activated Ca+2 influxes in BLA projection neurons	Karst et al. (2002)
Wistar rats; *Toxoplasma gondii* infection	Parallel decrease in BLA dendritic length and circulating baseline corticosterone	Mitra et al. (2013)
Wistar rats; BLA cytotoxic lesion	Reduction in innate and learned fear response to a live cat	Bindi et al. (2018)
Sprague-dawley rats; Disruption of cholinergic projection to the BLA	Reduced unconditioned fear to the car hair	Power and McGaugh (2002)

But the neuronal activation of BLA neurons during exposure does not separate incidental activation from involvement in the mediation of fear response. Predator odors do cause Fos expression and neuronal firing in a wide range of brain regions including the medial amygdala and other components of medial hypothalamic zones (Dielenberg et al., 2001). It is plausible that activation of BLA neurons relates more to Pavlovian conditioning rather than unconditioned fear itself (Takahashi et al., 2008). This possibility is shown by the observation that temporary or permanent lesions of BLA cause disruption of conditioned fear to cat odors in rats (Takahashi et al., 2007).

Nonetheless, several lesions studies show the possible role of BLA in unconditioned behavioral response to predators. Early resection studies of a medial temporal lobe in monkeys, for example, showed the development of “psychic blindness” in subjects, as a response, to stimuli which were loaded with aversion in non-manipulated animals (Lanska, 2018). Fiber sparing lesions of BLA reduces aversion to predator odors in rats in parallel to causing deficits in the conditioning process (Bindi et al., 2018). Disruption of cholinergic projections to the BLA in rats reduces unconditioned freezing to cat fur (Power and McGaugh, 2002). Similarly, optogenetic inhibition of anterior cingulate cortex projections to BLA potentiates freezing of mice to fox urine, and extraneous activation of this pathway reduces freezing (Jhang et al., 2018). These experiments suggest that BLA and its communication from upstream brain regions do change prey response to predator chemosensory cues. Apropos, BLA send moderate density of efferent fibers to medial amygdala. Anterograde tracer *Phaseolus vulgaris* leucoagglutinin travels to dorsal portion of medial amygdala in rats (Pitkänen et al., 1995). Sensory information reaches independently to basolateral and medial amygdala. It is plausible, and currently untested, that intra-amygdaloidal connections between basolateral and medial amygdala can allow an inter-dependent representation of the emotional valence in the predator associated olfactory cues.

Moreover, BLA might have a heterogeneous role in defensive behaviors dependent on specific predator odors used in the experiments. For example, chronic exposure to odor from ferret anal glands induces a set of physiological changes that are similar to those of chronic stress with robust BLA involvement (Campeau et al., 2008). This includes atrophy of thymus and hypertrophy of adrenal glands. Odors from ferret anal glands, and cat urine also activate BLA neurons apart from other brain regions in medial hypothalamic system (Campeau et al., 2008; Govic and Paolini, 2015). A reversible lesion of BLA reduces unconditioned fear to cat fur (Vazdarjanova et al., 2001). Optogenetic inactivation of anterior cingulate cortex, a brain region with selective efferent reaching BLA, enhances unconditioned fear to fox urine (Jhang et al., 2018). In contrast, several studies fail to find significant effects of BLA lesions on unconditioned defensive behaviors upon exposure to 2,3,5-trimethyl-3-thiazoline (Müller and Fendt, 2006; Rosen et al., 2008); a component of fox fecal odor. The difference between 2,3,5-trimethyl-3-thiazoline and other predator odors suggest a disparity in how these heterospecific cues are processed by BLA. Alternatively, it is possible that 2,3,5-trimethyl-3-thiazoline is perceived as a noxious odor rather than a predator related semiochemical (Fendt and Endres, 2008) and thus does not activate brain regions like BLA or medial hypothalamic zone.

## The Relationship Between BLA and Allostatic Load During Repeated Stressors

Exposure to predators induce physiological activation of stress endocrine axis through activation of hypothalamus-pituitary-adrenal axis (H-P-A axis; McEwen, 1998). Adrenal stress hormones include epinephrine from adrenal medulla and glucocorticoids from the adrenal cortex. Adrenal hormones prepare the body for metabolic demands placed by the need to fight-or-flight from the predators. Stress endocrine axis returns to the baseline once acute predator event has been successfully resolved (or the prey has been eaten, in which case the issue becomes moot!).

The homeostatic process works very well regarding switching the body between with-predator and san-predator episodes as far as such encounter are few and far between. Yet repeated or prolonged activation of stress endocrine axis takes a toll on neural mechanisms that control stress endocrine response through wear and tear (McEwen, 1998). Epinephrine does not readily cross the blood-brain barrier. But glucocorticoids can cross the barrier and bind to their receptors within a wide variety of brain regions. Central glucocorticoid signaling is important in maintaining the homeostatic regulation of stress hormones during acute events. For example, occupied glucocorticoid receptors within hippocampus lead to negative feedback on further release of adrenal hormones thus returning the physiological landscape to pre-stress baselines (Herman et al., 2003; Jankord and Herman, 2008). Yet the same neural machinery undergoes wear and tear during chronic stress; lowering the negative feedback, increasing glucocorticoids, exacerbating damage in the brain, further reducing homeostatic control. Thus, the relation between brain and stress hormone undergoes a drastic alteration from a situation of the sporadic stress of predator presence to a landscape dominated by frequent exposure to predators or cues of their presence. This reflects non-consumptive effects brought about by an allostatic load of repeated cycles of stress endocrine activation. BLA has a crucial role in this alteration.

**Figure 1:**
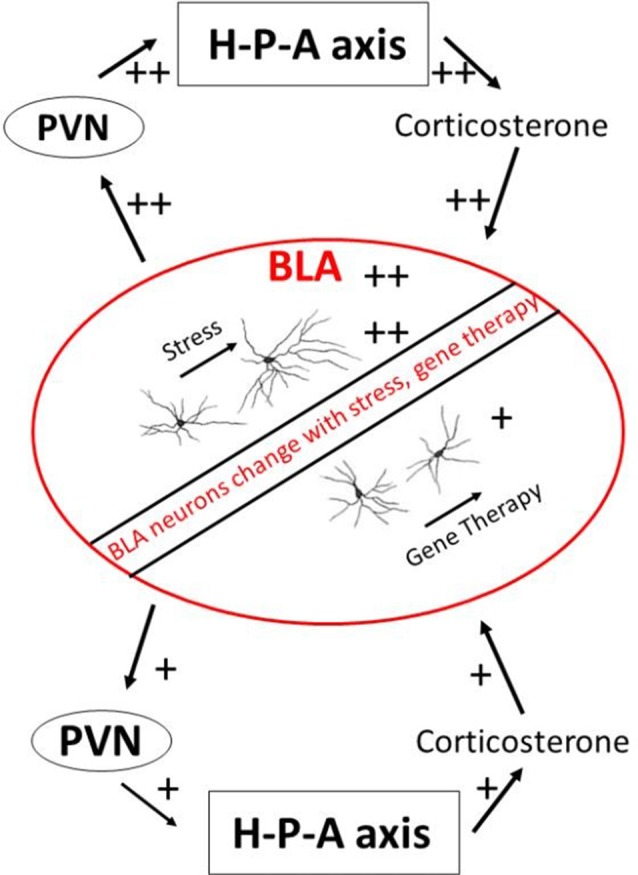
A schematic view of the BLA and stress endocrine response. BLA, basolateral amygdala; H-P-A axis, hypothalamus-pituitary-adrenal axis; PVN, paraventricular nucleus of hypothalamus.

BLA neurons undergo structural plasticity during stressful episodes (Vyas et al., 2002, 2003; Mitra et al., 2005). BLA contains a robust number of glucocorticoid receptors which bind to circulating stress hormones (Joëls and Karst, 2012). These receptors are important in creating long-lasting allostatic load. This is borne out by experiments involving exogenous supplementation of glucocorticoids, which elicits long-lasting dendritic expansion and spinogenesis in projection neurons of the BLA (Mitra and Sapolsky, 2008). Dendritic remodeling of BLA neurons can be prevented when experimental opportunities are created for competitive binding away from endogenous glucocorticoid receptors (Mitra et al., 2009c; Mitra and Sapolsky, 2010b). Similarly, exogenous glucocorticoids increase voltage-dependent calcium currents within individual BLA neurons (Karst et al., 2002; Joëls and Karst, 2012). This is important because calcium currents facilitate the formation of long-term potentiation and persistent synaptic changes. High levels of glucocorticoids also increase excitatory glutamatergic transmission within BLA, especially when it is coupled with prior exposure to epinephrine (Karst and Joëls, 2016). These studies collectively argue that chronic or severe stress leaves long-lasting footprints within the structure and function of BLA neurons (Figure 1). It is plausible that glucocorticoid-induced changes in BLA dendritic structure lead to greater excitability of BLA neurons through increasing number of synapses on the BLA neurons. Greater excitability of BLA neurons could, in turn, also increase dendritic complexity of BLA neurons through greater availability of calcium ions. The relationship between structure and electrophysiology remains unresolved at present.

These long-lasting footprints can change the response of animals to subsequent predator encounter. Several correlative studies point towards this direction. Rats show significant inter-individual variations in terms of long-term behavioral change resulting from acute exposure to a predator (Adamec and Shallow, 1993). Some individuals develop persistent anxiety, while anxiety in others remains undifferentiated from controls after a few weeks. Animals that show recalcitrant anxiety to cat presence in this paradigm have larger and more complex BLA dendritic trees compared to animals that do not show anxiogenesis (Mitra et al., 2009a). Similarly, the dendritic complexity of BLA neurons shows a predictive association with basal levels of circulating glucocorticoids in animals previously exposed to predator odor (Hegde et al., 2017). These studies are limited in the sense that they cannot be used to create cause-and-effect relationships. For example, it remains unclear if the BLA dendritic architecture is a primary change leading to change in glucocorticoids or change in behavior. Or if glucocorticoids lead to a parallel change in behavior and dendritic architecture through independent means. Nonetheless, these studies show that long-term non-consumptive consequences of predator exposure on stress hormones, behavioral change, and BLA structure coelute with each other.

There is an added possibility that changes within the BLA caused by stress hormones can change future secretion of stress hormones themselves through the effects of BLA on downstream paraventricular hypothalamic nucleus responsible for stress endocrine activation. Some paraventricular hypothalamus cells do receive direct projects from BLA, although these projections are sparse (Herman et al., 2003; Jankord and Herman, 2008). Yet there are at least two known polysynaptic pathways that lead from BLA to the paraventricular hypothalamus and thus to downstream modulation of stress hormones. The first pathway encompasses excitatory synapses made by BLA neurons on the central and medial amygdala, which then project to bed nucleus of stria terminalis and onward to the paraventricular hypothalamus (Dong et al., 2001). A smaller efferent route also leads from BLA directly onto bed nucleus of stria terminalis. These connections suggest that a change in BLA structure or function by stress can amplify response during subsequent exposure to stress, e.g., successive predator exposures. Some evidence of such metaplastic response comes from studies of BLA ablation. Lesions of BLA does not cause changes in basal glucocorticoids or those induced during acute stressors (Feldman et al., 1994). But inactivation of BLA causes excess secretion of adrenal glucocorticoids after a novel stressor (Bhatnagar et al., 2004). Studies have also directly compared effects of reducing excitation in BLA neurons on the secretion of adrenal steroids. A spatially-constrained over-expression of SK2 potassium channels within BLA, which should increase inhibition, leads to reduced glucocorticoids secretion in the periphery (Mitra et al., 2009c). Similarly, competitively blocking binding of glucocorticoid receptors within BLA results in lower stress hormone secretion from adrenals (Mitra et al., 2009b; Mitra and Sapolsky, 2010a). These observations point to an exciting possibility that changes within BLA caused by stress and changes to stress hormone axis caused by BLA represent a positive feedback loop that can rapidly change the metabolic state of animals during repeated predator encounters.

Exposure to ferret odor in early juvenile period increases social play in female rats but decreases play in males, when measured 3 weeks later (Stockman and McCarthy, 2017). Exposure to cat cues also lead to more risk assessment, higher avoidance and greater activity-suppression in female rats than males (Shepherd et al., 1992). Overexpression of corticotropin-releasing hormone during development also leads to gender-specific effects on response to presence of a predator. Under basal conditions, without overexpression of corticotropin-releasing hormone, only female mice show greater emotional reactivity after exposure to a cat (Toth et al., 2016). This gender difference is obliterated by overexpression of corticotropin-releasing hormone. These studies suggest substantial sexual dimorphism in effects of predator exposure on later behavior. Similarly, stress experienced by predator cues can interact with physiological state of the animals. For example, exposure to cat litter at start of inactive phase of the diurnal cycle causes more pronounced changes in behavior of rats compared to the same stressor at start of active phase (Cohen et al., 2015).

## Role of the BLA in Mediation of Environment-Dependent Behavioral Flexibility

BLA, apart from its place in allostatic change, is also involved in matching defensive behaviors to changing environments.

Several studies have used cues of environmental adversity to study the relationship between BLA and plasticity in the fear response. Early postnatal life has often been used in these studies because it represents a critical period of brain development and behavioral flexibility. Fear to predators in rat pups emerges around postnatal day 10, a period that is similar to the emergence of stress hormone secretion and amygdala development (Moriceau et al., 2004). Exogenous glucocorticoids at postnatal day 8 are sufficient to atypically cause the emergence of fear to a potential predator and activity within the BLA. Similarly, removal of adrenals and thus loss of glucocorticoids at postnatal day 12 prevents BLA activation and fear to predator usually present at this age (Moriceau et al., 2004). This process of fear emergence through glucocorticoid recruitment of BLA can be buffered through parental presence (Moriceau et al., 2006). Maternal presence attenuates stress endocrine response in early postnatal window resulting in a period where aversive Pavlovian conditioning procedures paradoxically lead to approach rather than avoidance of conditioned stimuli in an amygdala-dependent manner.

A similar interaction effect can also be seen when pregnant mice mothers are exposed to predator odors and offsprings are tested. Offsprings of predator odor-exposed mothers show enhanced fear to cat urine. Offsprings from predator odor-exposed mothers also show more robust secretion of adrenal glucocorticoids when they are exposed to a novel predator odor (St-Cyr and McGowan, 2015). These trans-generational effects occur concomitantly with increased mRNA abundance for corticotrophin-releasing hormone receptor in at least female offspring of predator odor-exposed mothers. The functional role of greater corticotrophin hormone receptor transcripts is not clear in this case. Yet sub-threshold doses of urocortin, an agonist for corticotrophin-releasing hormone, placed specifically within the BLA causes a long-lasting increase in anxiety and lowered neuronal inhibition in adult rats (Rainnie et al., 2004). These observations suggest that BLA and glucocorticoids can plausibly alter the magnitude of defensive responses elicited by animals as a function of predator density seen by the parents during critical developmental windows. Defensive responses can be entrained from parental experience to build anticipatory environment-dependent changes in the behavior through the interaction of BLA and glucocorticoids.

A reverse corollary can also be found whereby living in a sensorily enriched environment can dampen the defensive responses through proximate mechanisms based in the BLA. Relatively short periods of environmental enrichment in rats causes dendritic retraction in BLA neurons (Ashokan et al., 2016; Koe et al., 2016), a phenomenon that is opposite to dendritic expansion observed during exogenous glucocorticoid exposure. Similarly, this treatment reduces responsivity of stress endocrine axis. Interestingly the same enrichment paradigm also causes an increase in active risk assessment sorties made by rat towards cat urine (Mitra and Sapolsky, 2012). Cause and effect relationships in these studies remain under-investigated.

## Conclusion

Exposure to predators cause both consumptive and non-consumptive consequences for the prey animals. BLA is likely an important mediator of non-consumptive effects of predation on prey behavior. Exposure to predator often leaves a historical trace in the functioning of the stress endocrine axis. BLA is a crucial node in proximate mechanisms of these effects through its reciprocal interaction with adrenal glucocorticoids. The strength of neuroendocrine interactions between BLA and adrenal hormones can be further modulated by the incipient environment. Exposure to predators can also initiate anticipatory investment in defensive behaviors. There is growing evidence that BLA and glucocorticoids are involved in this process. Further experiments are warranted to dissociate incidental relationship from causal sequences.

Stress generated from exposure to predators or predator cues have been used to create animal models of fear-related psychiatric disorders like post-traumatic stress disorder and anxiety disorders (Adamec et al., 2008; Zoladz and Diamond, 2016). Several observations suggest an important role of BLA in precipitation of symptoms in preclinical models. For example, excitatory neurotransmission within BLA is required for anxiety-like symptoms and concurrent neural activation in rats after exposure to a live cat (Adamec et al., 2005; Blundell and Adamec, 2007). Similarly, high-frequency BLA stimulation reduces anxiety-like behavior in rats after exposure to cat urine (Dengler et al., 2018); an effect similar to informational lesion observed in deep-brain stimulation paradigms. Relationship between predator exposure, BLA and psychiatric conditions remains currently understudied; and represents an important opportunity to understand clinically abnormal fear in the future.

## Author Contributions

RM contributed to the organization, literature search and writing of this manuscript.

## Conflict of Interest Statement

The author declares that the research was conducted in the absence of any commercial or financial relationships that could be construed as a potential conflict of interest.
